# Avaliação Prognóstica da Microalternância da onda T na Cardiomiopatia Hipertrófica em um Seguimento Clínico de 9 anos

**DOI:** 10.36660/abc.20220833

**Published:** 2023-08-18

**Authors:** Murillo Oliveira Antunes, Edmundo Arteaga-Fernandez, Nelson Samesima, Horácio Gomes Pereira, Afonso Yoshikiro Matsumoto, Richard L. Verrier, Carlos Alberto Pastore, Charles Mady

**Affiliations:** 1 Universidade de São Paulo Faculdade de Medicina Hospital das Clínicas São Paulo SP Brasil Instituto do Coração do Hospital das Clínicas da Faculdade de Medicina da Universidade de São Paulo, São Paulo, SP – Brasil; 2 Universidade São Francisco Bragança Paulista SP Brasil Universidade São Francisco, Bragança Paulista, SP – Brasil; 3 Fleury Group São Paulo SP Brasil Fleury Group, São Paulo, SP – Brasil; 4 Beth Israel Deaconess Medical Center Boston EUA Beth Israel Deaconess Medical Center, Boston – EUA

**Keywords:** Cardiomiopatia Hipertrófica, Morte Súbita, Arritmias Cardíacas, Desfibriladores Implantáveis

## Abstract

**Fundamento:**

A morte súbita cardíaca (MSC), decorrente de arritmias ventriculares, é a principal complicação da cardiomiopatia hipertrófica (CMH). A microalternância da onda T (MAOT) está associada à ocorrência de arritmias ventriculares em diversas cardiopatias, mas seu papel na CMH permanece incerto.

**Objetivo:**

Avaliar associação da MAOT com a ocorrência de MSC ou arritmias ventriculares malignas em pacientes com CMH.

**Método:**

Pacientes com diagnóstico de CMH e classe funcional I-II (NYHA) foram selecionados de forma consecutiva. No início do seguimento os participantes realizaram a avaliação da MAOT pela metodologia da média móvel modificada no teste de esforço. Os resultados foram classificados em alterado ou normal. O desfecho foi composto por MSC, fibrilação ventricular, taquicardia ventricular sustentada (TVS) e terapia apropriada do cardioversor desfibrilador implantável (CDI). O nível de significância estatística foi de 5%.

**Resultados:**

Um total de 132 pacientes (idade média de 39,5±12,6 anos) foram incluídos, com tempo de seguimento médio de 9,5 anos. A MAOT foi alterada em 74 (56%) participantes e normal em 58 (44%). Durante o seguimento, nove (6,8%) desfechos ocorreram, com prevalência de 1,0%/ano, sendo seis casos de MSC, dois choques apropriados do CDI e um episódio de TVS. MAOT alterada foi associada à taquicardia ventricular não sustentada no Holter (p=0,016), espessura septal≥30 mm (p<0,001) e resposta inadequada da pressão arterial ao esforço (p=0,046). Cinco pacientes (7%) e quatro pacientes (7%) com MAOT alterada e normal, respectivamente, apresentaram desfecho primário [OR=0,85(IC95%: 0,21–3,35, p=0,83)]. Curvas de eventos de Kaplan-Meir não apresentaram diferenças entre MAOT normal e alterada.

**Conclusão:**

A MAOT alterada não foi associada à ocorrência de MSC ou arritmias ventriculares potencialmente fatais em pacientes com CMH, e a baixa taxa desses eventos em um seguimento em longo prazo sugere o bom prognóstico dessa cardiopatia.

**Figure:**
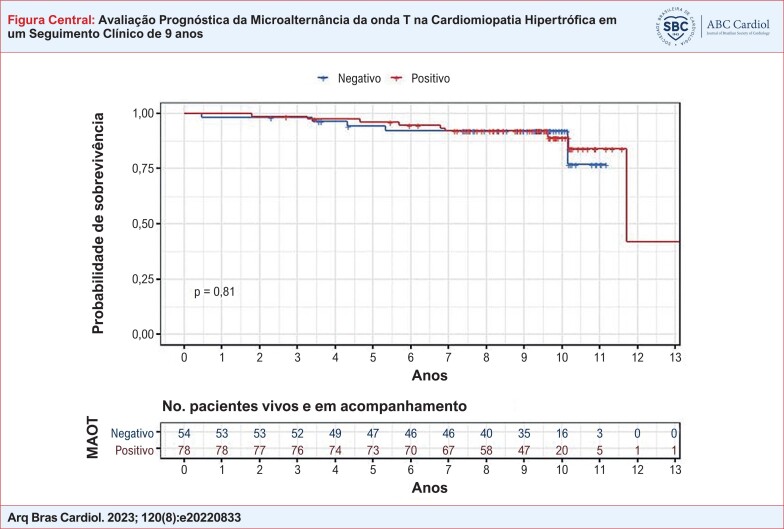
Curva de Kaplan-Meier para sobrevivência livre de eventos (morte súbita cardíaca arrítmica, parada cardíaca ressuscitada, ocorrência de fibrilação ventricular ou taquicardia ventricular sustentada e/ou ocorrência de terapia apropriada de desfibrilação do cardioversor desfibrilador implantável com base no teste de microalternância da onda T (MAOT). MAOT: microalternância da onda T.

## Introdução

A Cardiomiopatia Hipertrófica (CMH) é a mais prevalente das cardiopatias de transmissão genética e constitui a principal causa de morte súbita (MSC) em jovens e atletas.^1,2^ A MSC causada pela ocorrência de fibrilação ventricular (FV), precedida ou não por episódio de taquicardia ventricular (TV), é prevenida e tratada com implante do cardioversor desfibrilador implantável (CDI).^3–5^

O substrato miocárdico que predispõe às arritmias ventriculares na CMH inclui hipertrofia e desarranjo dos cardiomiócitos, com formação de fibrose intersticial. Essa, associada à isquemia miocárdica transitória durante alta frequência cardíaca e obstrução dinâmica da via de saída do ventrículo esquerdo (VE), aumentam a vulnerabilidade às arritmias malignas. Porém, devido à ampla diversidade de apresentações desse substrato na prática clínica, é um desafio reconhecer com precisão os indivíduos com maior risco de MSC que se beneficiariam com o CDI.^6–8^ Assim, torna-se necessária a descoberta de novos métodos diagnósticos para estratificar o risco de MSC, possibilitando uma terapêutica individualizada e custo efetivo para esses pacientes.

A microalternância da onda T (MAOT) consiste na variação microscópica da amplitude ou da morfologia da onda T em cada batimento cardíaco, refletindo heterogeneidade da repolarização ventricular, e sua avaliação é comumente realizada nesse contexto.^9,10^ A presença e magnitude da MAOT estão associadas às condições predisponentes para o início e perpetuação de arritmias ventriculares. A MAOT é um marcador de risco para arritmias ventriculares em pacientes com cardiomiopatia, isquemia coronariana e síndromes relacionadas a arritmias hereditárias, mas em pequenas coortes não teve consistência como preditor de risco na CMH.^11–14^

Assim, o objetivo do presente estudo foi avaliar se a MAOT está associada à ocorrência de arritmias potencialmente fatais ou MSC arrítmica em pacientes com CMH.

## Método

### Casuística

Estudo prospectivo realizado entre janeiro de 2010 a dezembro de 2019. Nós selecionamos pacientes de forma consecutiva no ambulatório de Miocardiopatias Gerais do Instituto do Coração do Hospital das Clínicas da Faculdade de Medicina da Universidade de São Paulo (InCor-HC- FMUSP) no período de abril de 2010 a junho de 2013.

No momento da inclusão todos os participantes realizaram o exame de MAOT. Nenhuma medicação foi descontinuada para realização dos exames, prezando pela segurança dos participantes, pois, principalmente nas formas obstrutivas, o esforço físico piora o gradiente na via de saída do VE podendo ocasionar hipotensão e/ou síncope.

O estudo foi aprovado pelo Comitê de Ética em Pesquisa do Hospital das Clínicas da Faculdade de Medicina da Universidade de São Paulo sob número 0665/09, e todos os participantes assinaram o termo de consentimento livre e esclarecido.

### Critérios de inclusão

Foram incluídos pacientes de ambos os sexos, com idade acima de 18 anos e diagnóstico de CMH confirmado pelo ecocardiograma bidimensional. Esse foi definido pela presença de espessura parietal ≥15 mm em qualquer segmento do VE (ou ≥13 mm para os indivíduos com parentes de primeiro grau portadores de CMH),^3^ independente da presença de gradiente obstrutivo na via de saída do VE, e ausência de doenças cardíacas ou sistêmicas que poderiam causar hipertrofia ventricular esquerda. Os participantes deveriam estar em classe funcional (CF) I-II da *New York Heart Association* (NYHA) e ter condições físicas para conseguir realizar o exercício em esteira.

### Critérios de exclusão

Os critérios de exclusão foram classe funcional III ou IV da NYHA, presença de dilatação do VE (diâmetro diastólico > 60 mm) e/ou disfunção sistólica (fração de ejeção < 0,50), terapia prévia de redução septal (miectomia ou alcoolização septal), fibrilação atrial, doença coronariana crônica, hipertensão arterial, diabetes mellitus tipo 1 ou 2, doença renal crônica (estágio III-V) e valvopatias primárias.

### Avaliação da MAOT

O exame da MAOT foi realizado em esteira rolante da marca General Eletric (GE), modelo CASE 6.5, utilizando o protocolo de esforço Naughton modificado, devido às suas características atenuadoras que reduzem o aparecimento de ruídos e artefatos que interferem na análise do exame. O esforço em esteira rolante foi realizado, pois o aumento da frequência cardíaca amplifica a magnitude da alternância da onda T.^15,16^

Os valores da MAOT foram calculados de forma contínua e automática, pela média móvel modificada (MMM). Resumidamente, o algoritmo da MMM separa as batidas ímpares das pares; as morfologias médias dos batimentos pares e ímpares são calculadas separadamente e continuamente atualizadas por um fator de ponderação de 1/8 da diferença entre a média atual e os novos batimentos recebidos. A atualização é calculada para cada batida recebida e resulta em médias móveis contínuas das batidas ímpares e pares. Os valores de MAOT foram calculados continuamente durante todo o teste de esforço, com as medidas atualizadas a cada 15 segundos da monitorização, avaliadas nas 12 derivações eletrocardiográficas, durante todo o exame (repouso, exercício e recuperação).^15,17^ As derivações que apresentaram valores de ruído acima de 20 μV foram excluídas. O exame foi considerado alterado na presença de valores da MAOT ≥53 μV,^1^ observada em qualquer derivação eletrocardiográfica.^15^

Todos os exames foram revisados por um único observador que era cego aos dados clínicos dos pacientes.

### Classificação de Risco para MSC

Os participantes foram classificados em grupos de alto ou baixo risco para ocorrência de MSC conforme as orientações da diretriz de CMH da *American College of Cardiology Foundation*/AHA 2020.^3^ Desta forma, participantes que apresentavam um ou mais dos seguintes fatores de risco foram classificados como alto risco: antecedente pessoal de parada cardiorrespiratória ou taquicardia ventricular sustentada (TVS), espessura ventricular ≥ 30 mm em qualquer segmento, história familiar de MSC em parentes de primeiro grau com idade inferior a 50 anos, episódio recente de síncope com suspeita de causa arrítmica, aneurisma apical de VE e fração de ejeção do VE (FEVE) < 50%.^3^

### Seguimento e desfechos dos estudos

O acompanhamento clínico foi realizado com intervalos de quatro semanas, por meio de consultas médicas presenciais ou contato telefônico para os pacientes que não compareceram ao retorno médico.

O desfecho primário foi MSC, parada cardíaca ressuscitada secundária à FV ou à TV, episódio de FV ou TVS ou ocorrência de terapia apropriada com CDI.

A MSC foi definida como colapso súbito e inesperado ocorrendo dentro de 1 hora do início dos sintomas em pacientes clinicamente estáveis, resultando em óbito em até 24 horas do início da apresentação. TVS foi definida por evento com um mínimo de três complexos ventriculares e duração superior a 30 segundos. Para os eventos ocorridos fora da nossa instituição, as circunstâncias do óbito foram determinadas por entrevista por telefonema com um familiar ou relatórios médicos fornecidos pelos serviços que assistiram o paciente. Qualquer desacordo nas informações do evento foi discutido com três médicos cardiologistas e decidido por consenso.

### Análise estatística

O cálculo do tamanho amostral considerando poder estatístico de 85%, tamanho de efeito de 0,60 e nível de significância de p < 0,05, foi de mínimo da amostra 102 indivíduos, 51 por grupo.

As análises descritivas para variáveis quantitativas paramétricas estão apresentadas por média e desvio padrão, as não paramétricas por mediana e intervalo interquartil, enquanto as variáveis categóricas estão descritas por frequências absolutas (n) e relativas (%).

Para a avaliação de distribuição normal dos dados, utilizou-se o teste de Kolmogorov-Smirnov. Na avaliação dos dados com distribuição normal, utilizou-se o teste T não-pareado para os dados paramétricos e teste de Mann-Whitney para os dados não paramétricos. O teste de Fisher foi utilizado para comparação das variáveis categóricas entre os grupos. A curva de Kaplan-Meier foi empregada para avaliar as taxas de eventos dos participantes em relação ao resultado do exame da MAOT.

A taxa de mortalidade total foi calculada pela divisão do número total de pacientes que foram a óbito pelo número de pacientes estudados. A taxa de mortalidade anual foi obtida pela divisão da taxa de mortalidade total pelo tempo de acompanhamento médio.

Foram calculados *odds ratio* (OR), sensibilidade, especificidade e acurácia para avaliação da ocorrência do desfecho primário em relação à MAOT alterada e à classificação de risco clínico.

Todos os testes foram bicaudais e um nível de significância de 5% foi adotado. Os cálculos foram realizados com auxílio do software R 3.6.0^18^ e os gráficos foram construídos com o pacote ggplot2.^19^

## Resultados

Participaram do estudo 132 pacientes com idade média de 39,5 anos e acompanhados por tempo médio 9,5 anos (intervalo interquartil 4,7 - 10,1 anos). Durante o estudo não tivemos nenhuma perda de seguimento de participantes. A descrição da amostra encontra-se na Tabela 1.

**Tabela 1:** Características dos pacientes

Características		Total (n = 132)	Resultado da MAOT		p-valor
			Normal (n = 58)	Alterada (n = 74)	
**Idade**		39,5 ± 12,6	39,0 ± 11,7	40,3 ± 12,8	0,166
**Sexo - Masculino**		85 (64,4%)	38 (70,4%)	47 (60,3%)	0,270
**Sobrevida (anos)**		9,456[7,91;10,0]	9,59[7,88;10,0]	9,42[7,84;9,4]	0,342(1)
**Medida MAOT, μV**		76 ± 54,0	32 ± 12,0	110 ± 50,0	**<0,000**
**Desfecho Primário** #		9 (6,8%)	4 (7,0%)	5 (7,0%)	0,824
	MSC	06	02	04	NA
	Choque CDI	02	01	01	NA
	Ocorrência TVS	01	01	00	NA
**Ecocardiograma, mm**					
	Septo	23,0 [20,0; 27,0]	23,0 [20,0; 27,0]	24,5[20,7; 30,2]	0,532(1)
	Átrio esquerdo	42,6 ± 6,99	41,9 ± 7,11	43,2 ± 6,91	0,923
	Ventrículo esquerdo	4,1 ± 5,39	43,2 ± 4,48	43,1 ± 5,97	0,117
	Fração de ejeção, %	72,1 ± 8,07	72,3 ± 7,75	72,1 ± 8,34	0,628
**Forma obstrutiva**		35 (26,5%)	14 (25,9%)	21 (26,9%)	0,531
**Fatores de Risco MSC**					
	Síncope	12 (9,1%)	2 (3,7%)	10 (12,8%)	0,073
	TVNS no Holter	37 (28,0%)	9 (16,7%)	28 (35,9%)	**0,016**
	HF de MSC	27 (20,5%)	7 (13,0%)	20 (25,6%)	0,076
	Septo ≥ 30 mm	27 (20,5%)	3 (5,6%)	24 (30,8%)	**<0,001**
	Queda PAS no TE	19 (14,4%)	4 (7,4%)	15 (19,2%)	**0,046**
**AHA/ACC 2020** *					
	Alto Risco	70 (53,0%)	22 (28%)	58(72%)	**<0,001**
**Medicação**					
	Betabloqueador	95 (72,0%)	44(81,5%)	51 (65,4%)	0,043
	Bloqueador Canais Ca^+^	16 (12,1%)	5 (9,3%)	11 (14,1%)	0,485
	Amiodarona	18 (15,1%)	2 (4,1%)	16 (22,9%)	0,012

(1) Teste de Mann-Whitney, mediana e intervalos entre quartis. NA: Não avaliado.

# Desfecho primário composto por Morte Súbita Cardíaca (MSC), ou parada cardíaca ressuscitada, ou ocorrência de fibrilação atrial ou taquicardia ventricular sustentada (TVS), e ou terapia apropriada de desfibrilação do cardioversor desfibrilador implantável (CDI).

* A diretriz AHA/ACC 2020 define como pacientes de alto risco na presença de um desses: história de MSC em parentes do primeiro grau com ≤ 50 anos, espessura de parede ≥ 30 mm, ≥ 1 episódios de síncope inexplicada, aneurisma apical e fração de ejeção <50%. HF: história familiar; TVNS: taquicardia ventricular não-sustentada; PAS: pressão arterial sistólica; TE: teste de esforço

**Tabela 2:** Pacientes que apresentaram o desfecho primário

Idade (anos)	Sexo	Resultado MAOT	Valor (μV)	Fatores risco	Indicação CDI#	Sobrevida (anos)	Desfecho
30	F	Alterada	185	0	Classe III (B)	1,8	MSC
28	M	Normal	19	0	Classe III (B)	3,4	MSC
31	M	Normal	20	Septo≥30 mm	Classe IIa (B)	5,3	MSC
24	M	Alterada	**56**	TVNS	Classe III (B)	6,8	MSC
56	M	Alterada	72	Septo≥30 mm	Classe IIa (B)	6,9	MSC
43	M	Alterada	**73**	HF, Septo≥30, TVNS	Classe IIa (B)	9,6	Choque CDI
36	F	Normal	27	HF	Classe IIa (B)	10,2	TVS
32	F	Alterada	**54**	Síncope, HF, TVNS	Classe IIa (B)	11,7	MSC
48	M	Normal	14	Síncope	Classe IIa (B)	10,2	Choque CDI

# Conforme orientação das diretrizes do *American College of Cardiology/American Heart Association* 2020; HF: história familiar de morte súbita cardíaca TVNS: taquicardia ventricular não-sustentada MSC: Morte súbita cardíaca CDI: cardioversor desfibrilador implantável; TVS: taquicardia ventricular sustentada; MAOT: microalternância da onda T

Durante o seguimento, ocorreram 12 óbitos, totalizando uma mortalidade geral de 1,3% ao ano; seis casos decorreram de MSC, quatro por insuficiência cardíaca avançada e dois por acidente vascular cerebral. Quanto à frequência de desfechos primários, seis pacientes sofreram MSC (ocorridos fora do hospital), dois choques apropriados do CDI e um episódio de TVS com prevalência de 1,0% ao ano.

A MAOT alterada foi associada com espessura septal aumentada, presença de taquicardia ventricular não-sustentada (TVNS) no Holter de 24h, queda da pressão no esforço e classificação da AHA/ACC de alto risco.

Diferentemente do esperado, os valores da MAOT foram menores no grupo que apresentou o desfecho primário quando comparado ao grupo sem eventos (59 ± 33 μV versus 79 ± 42 μV, p=0,352). As curvas de Kaplan-Meier livres de eventos não foram estatisticamente diferentes entre pacientes com e sem alteração da MAOT (Figura Central).

A MAOT, como a classificação de risco da AHA/ACC, não foi capaz de prever a ocorrência do desfecho primário, apresentando ambas baixas acurácias (Tabela 3).

**Tabela 3:** Risco, sensibilidade e especificidade para ocorrência do desfecho

		Desfecho			IC (95%)					
		Sim	Não	OR	Inferior	Superior	p	Sensibilidade	Especificidade	Acurácia
**MAOT**										
	Normal	4	54	0,85	0,21	3,3	0,83	55,6%	43,9%	44,7%
	Alterada	5	69							
**Diretrizes AHA 2020**										
	Baixo Risco	2	60	3,3	0,66	16,8	0,12	77,8%	48,8%	50,8%
	Alto Risco	7	63							

Desfecho composto por: morte súbita cardíaca, parada cardíaca ressuscitada, episódio de fibrilação ventricular ou taquicardia ventricular sustentada e/ou ocorrência de terapia apropriada do cardioversor desfibrilador implantável; AHA: *American Heart Association*; OR: *Odds Ratio*; IC: intervalo de confiança.

## Discussão

O objetivo do presente estudo foi avaliar o valor prognóstico da MAOT em pacientes com CMH. Os nossos resultados demonstraram que, durante o esforço, os pacientes com CMH apresentavam valores mais altos de MAOT, com média 76±54 μV que os descritos em outras cardiopatias.^20–22^ Porém, no presente estudo, o exame de MAOT alterado não apresentou associação com a ocorrência de arritmias ventriculares potencialmente fatais em pacientes com CMH.

Os valores elevados da MAOT encontrado nessa população é explicada pela presença do substrato arritmogênico típico da CMH. Tal substrato é composto por hipertrofia e desarranjo arquitetural dos cardiomiócitos, associados à formação de fibrose intersticial difusa,^1,23,24^ resultando em alternâncias de repolarização nos cardiomiócitos e maior magnitude da MAOT.

Ainda, a extensão da hipertrofia miocárdica associa-se com um aumento da magnitude da MAOT, o que também foi encontrado em nossa população. A MAOT alterada associou-se significativamente com maior espessura septal, corroborando com os achados descritos por Puntmann et al.^25^

MAOT alterada relaciona-se com mecanismos de reentrada e FV.^9–11^ Os nossos achados sugerem que na CMH, outros mecanismos arritmogênicos estão envolvidos na origem das arritmias ventriculares e MSC, pois a maioria dos indivíduos apresentavam valores altos de MAOT, mas taxa de desfechos durante o seguimento foi baixa (1,0%/ano).

Fuchs et al. ^14^ também demonstraram que MAOT não foi útil para prever MSC. Como a MAOT não é um fenômeno estático e pode modificar-se ao longo do tempo, sugere-se que, durante a evolução da doença, indivíduos com resultado normal possam desenvolver valores anormais da alternância de onda T.

Atualmente, o implante do CDI na CMH é indicado a pacientes com fatores de riscos clínicos e classificados com alto risco, seguindo-se as diretrizes da AHA/ACC.^3^ No presente estudo, a MAOT alterada foi associada com fatores de risco para MSC e foi sensível para identificar os indivíduos classificados pela diretriz como de alto risco para MSC. Entretanto, na nossa coorte, a MAOT e a diretriz da AHA/ACC falharam em identificar os indivíduos que apresentaram arritmias ventriculares potencialmente fatais ou alto risco para MSC. Assim, como também descrito por Freitas et al.,^26^ os nossos achados demonstraram que os critérios da AHA/ACC apresentam baixa acurácia para identificar os indivíduos que necessitam do CDI. Desta forma, acreditamos que sejam necessários novos estudos, multicêntricos e nacionais, para avaliar a acurácia da indicação do CDI para a população do Brasil, atualmente baseada em resultados obtidos de estudos de outros países.

### Limitações do estudo

O presente estudo apresenta várias limitações. A baixa taxa de desfechos durante o seguimento reduz o poder do estudo, favorecendo a ocorrência de erro estatístico tipo II. A realização do estudo em centro único está sujeita a viés de seleção dos participantes. Sabe-se que alguns medicamentos, como drogas beta-adrenérgicas,^17,27,28^ bloqueadores dos canais de sódio^29^ e amiodarona diminuem a magnitude da MAOT, e o uso de medicações por nossos pacientes não foi suspenso para realização do exame. Além disso, como a documentação do ritmo não estava disponível para todos os casos de MSC, algumas dessas mortes podem ter sido de natureza não arrítmica.

## Conclusão

Os resultados deste estudo mostraram que a MAOT alterada não está associada a ocorrência de MSC e/ou arritmias ventriculares malignas em pacientes com CMH. As baixas taxas de eventos observados durante o seguimento demonstram o caráter benigno dessa cardiopatia, com baixa taxa de mortalidade e expectativa de vida normal.
