# Supplementary material for: Avaliação Prognóstica da Microalternância da onda T na Cardiomiopatia Hipertrófica em um Seguimento Clínico de 9 anos
Source: Arq Bras Cardiol. 2023 Aug 18;120(8):e20220833. [Article in Portuguese] doi: 10.36660/abc.20220833 (PMC10519242; doi:10.36660/abc.20220833)
Supplement: Translation

# Translation: Prognostic Evaluation of Microvolt T-Wave Alternans in Hypertrophic Cardiomyopathy: 9-year Clinical Follow-up

**Authors:** Murillo Oliveira Antunes, Edmundo Arteaga-Fernandez, Nelson Samesima, Horácio Gomes Pereira, Afonso Yoshikiro Matsumoto, Richard L. Verrier, Carlos Alberto Pastore, Charles Mady

**Affiliations:** 1 São Paulo SP Brasil Instituto do Coração do Hospital das Clínicas da Faculdade de Medicina da Universidade de São Paulo, São Paulo, SP – Brasil; 2 Bragança Paulista SP Brazil Universidade São Francisco, Bragança Paulista, SP – Brazil; 3 São Paulo SP Brazil Fleury Group, São Paulo, SP – Brazil; 4 Boston USA Beth Israel Deaconess Medical Center, Boston – USA

**Keywords:** Cardiomyopathy, Hypertrophic, Death, Sudden, Arrhythmias, Cardiac, Defibrillators, Implantable

## Abstract

**Background:**

Sudden cardiac death (SCD) resulting from ventricular arrhythmia is the main complication of hypertrophic cardiomyopathy (HCM). Microvolt T-wave alternans (MTWA) is associated with the occurrence of ventricular arrhythmias in several heart diseases, but its role in HCM remains uncertain.

**Objective:**

To evaluate the association of MTWA with the occurrence of SCD or potentially fatal ventricular arrhythmias in HCM patients in a long-term follow-up.

**Methods:**

Patients diagnosed with HCM and NYHA functional class I-II were consecutively selected. At the beginning of the follow-up, the participants performed the MTWA evaluation using the modified moving average during the stress test. The results were classified as altered or normal. The composite endpoint of SCD, ventricular fibrillation, sustained ventricular tachycardia (SVT) or appropriate implantable cardiac defibrillation (ICD) therapy was assessed. The level of significance was set at 5%.

**Results:**

A total of 132 patients (mean age of 39.5 ± 12.6 years) were recruited and followed for a mean of 9.5 years. The MTWA test was altered in 74 (56%) participants and normal in 58 (44%). Nine events (6.8%) occurred during the follow-up, with a prevalence of 1.0%/year – six SCDs, two appropriate ICD shocks and one episode of (SVT). Altered MTWA was associated with non-sustained ventricular tachycardia on Holter (p = 0.016), septal thickness ≥30 mm (p < 0.001) and inadequate blood pressure response to effort (p = 0.046). Five patients with altered MTWA (7%) and four patients with normal MTWA (7%) had the primary outcome [OR = 0.85 (95% CI: 0.21 - 3.35, p=0.83)]. Kaplan-Meir event curves showed no differences between normal and altered MTWA.

**Conclusion:**

Altered MTWA was not associated with the occurrence of SCD or potentially fatal ventricular arrhythmias in HCM patients, and the low rate of these events during long-term follow-up suggests the good prognosis of this heart disease.

## Introduction

Hypertrophic cardiomyopathy (HCM) is the most prevalent genetically transmitted heart disease and is the main cause of sudden cardiac death (SCD) in athletes and young individuals.^1,2^ SCD caused by ventricular fibrillation (VF), preceded or not by ventricular tachycardia (VT), is prevented and treated with implantable cardioverter-defibrillator (ICD).^3–5^

The myocardial substrate that predisposes to ventricular arrhythmias in HCM includes cardiomyocyte hypertrophy and disarray, with formation of interstitial fibrosis. This, associated with transient myocardial ischemia during high heart rate and dynamic obstruction of the left ventricle, increases the vulnerability of malignant arrhythmias. However, due to the wide diversity of presentations of this substrate in clinical practice, it is challenging to accurately recognize individuals at higher risk of SCD that would benefit from an ICD.^6–8^ Therefore, new diagnostic methods for risk stratification of SCD are needed, allowing an individualized and cost-effective treatment of these patients.

Microvolt T-wave alternans (MTWA) consists of beat-to-beat microscopic fluctuation in the morphology or amplitude of the T-wave, reflecting the heterogeneity of ventricular repolarization, and its assessment is commonly performed in this context.^9,10^ The presence and the magnitude of MTWA are associated with predisposing conditions to the onset and perpetuation of ventricular arrhythmias. MTWA is a risk marker of ventricular arrhythmias in patients with cardiomyopathy, coronary ischemia, and syndromes related to inherited arrhythmias, but in small samples, but in small samples, MTWA was not consistent as a HMC risk predictor.^11–14^

**Figure:** Kaplan-Meier event-free survival curves for the outcomes – arrhythmic sudden cardiac death, resuscitated cardiac arrest, ventricular fibrillation or sustained ventricular tachycardia, or appropriate therapy with implantable cardioverter-defibrillator; MTWA: Microvolt T-wave alternans.

Thus, the aim of the present study was to assess whether MTWA is associated with the occurrence of potentially fatal arrhythmias or arrhythmic SCD in patients with HMC.

## Methods

### Patients

This was a prospective study conducted between January 2010 and December 2019. We selected consecutive patients at the cardiomyopathy outpatient clinic of the Heart Institute (InCor) of the University of São Paulo between April 2010 and June 2013.

All participants underwent the MTWA test at study inclusion. For the sake of the safety of participants, no medication was discontinued for the tests, since especially in the obstructive forms, physical exertion increases the left ventricular (LV) outflow tract gradient and may lead to hypotension and/or syncope.

The study was approved by the ethics committee of the Hospital das Clínicas da Faculdade de Medicina da Universidade de São Paulo (approval number 0665/09), and all participants signed a consent form.

### Inclusion criteria

Adult patients (>18 years of age) of both sexes, with a diagnosis of HCM confirmed by two-dimensional echocardiography were included in the study. This was defined as the presence of LV wall thickness ≥ 15 mm in any segment of the left ventricle (or ≥13mmm for individuals with first degree relatives with HCM),^3^ regardless of an obstructive gradient in the LV outflow track, and absence of cardiac or systemic disease that may cause LV hypertrophy. Participants would have New York Heart Association (NYHA) functional class (FC) I-II and be physically capable of exercising on a treadmill.

### Exclusion criteria

Exclusion criteria included NYHA FC III or IV, LV dilatation (diastolic diameter > 60mm) and/or systolic dysfunction (ejection fraction < 0.50), previous septal reduction therapy (septal myectomy or alcohol ablation), atrial fibrillation, chronic coronary artery disease, arterial hypertension, type 1 or type 2 diabetes mellitus, chronic renal disease (stage III-V) and primary valve diseases.

### Assessment of MTWA

The MTWA test was performed on a treadmill (General Electric CASE system, version 6.5) using a modified Naughton exercise protocol, as it minimizes noise and artifacts that affect the analysis of the test. Treadmill stress test was performed, as the increase in the heart rate increases the magnitude of T wave alternans.^15,16^

MTWA values were calculated automatically and continuously using the modified moving average (MMA). Briefly, the MMA algorithm separates odd beats from even ones; average morphologies of odd and even beats are calculated separately and continuously updated by 1/8 of the difference between current average and new incoming beats. The update for each beat is calculated, resulting in continuous moving average of odd and even beats. MTWA values were continuously calculated during the stress test, with measurements updated every 15 seconds of monitorization, assessed by 12-lead electrocardiogram during all the test (resting, exercise and recovery).^15,17^ The leads with noise levels >20 μV were excluded. The test was considered altered when MTWA values were ≥ 53 μV^1^ in any of the electrocardiographic leads.^15^

All tests were reviewed by the same observer who was blinded for the clinical data of the patients.

### Risk classification for SCD

Patients were classified into high-risk and low-risk groups for the occurrence of SCD according to the *American College of Cardiology Foundation* /AFA 2020 guidelines.^3^ Participants were classified at high risk when they had at least one of the following risk factors: previous cardiac arrest or sustained ventricular tachycardia (SVT), ventricular thickness ≥30 in any segment, family history of SCD in first-degree relatives younger than 50 years, recent episode of syncope caused by suspected arrhythmia, LV apical aneurysm, and LV ejection fraction (LVEF) < 50%.^3^

### Follow-up and outcomes

The clinical follow-up was conducted in intervals of four weeks by in-person visits or telephone calls to those patients who did not attend the visit.

The primary outcome was SCD, resuscitated cardiac arrest secondary to VF or VT, episode of VF or SVT, or appropriate therapy with ICD.

SCD was defined as a sudden and unexpected collapse in the first hour of the symptom onset in clinically stable patients, leading to death occurring within 24 h after the onset of the symptoms. SVT was defined as the occurrence of three or more ventricular complexes lasting longer than 30 seconds. For events occurring outside the institution, the circumstances of death were determined by telephone interviews with a family member or by data obtained from medical records provided by the health services where the patient was treated. Any disagreement between data related to the event was discussed by three cardiologists and resolved by consensus.

### Statistical analysis

Sample size was calculated considering a statistical power of 85%, effect size of 0.60, significance level of p<0.0, resulting in a minimum of 102 individuals, 51 per group.

Descriptive analyses of parametric quantitative variables were described as mean and standard deviation; the non-parametric variables were described as median and interquartile range, whereas the categorical variables were described as absolute (n) and relative (%) frequencies.

Normality of data distribution was assessed using the Kolmogorov-Smirnov test. In the analysis of normally distributed data, the unpaired t-test was used for parametric data and the Mann-Whitney test for non-parametric data. Fisher's exact test was used for comparison of categorical variables between the groups. The Kaplan-Meier curve was used to analyze the event rates of participants in relation to their MTWA results.

Overall mortality rate was calculated by dividing the number of patients who died by the number of patients studied. Annual mortality rate was calculated by dividing overall mortality rate by the average follow-up period.

To assess the occurrence of primary outcome and its relationship with altered MTWA and risk classification, we calculated odds ratio (OR), sensitivity, specificity, and accuracy.

All tests were two-tailed, and the level of significance was set at 0.05. Analyses were performed using the R software version 3.6.0^18^ and the graphs were constructed using the ggplot2.^19^

## Results

A total of 132 patients with mean age of 39.5 years were included and followed for a mean of 9.5 years (interquartile range of 4.7-10.1 years). There was no loss to follow-up. Description of the sample can be found in Table 1.

**Table 1:** Characteristics of the patients

Characteristics		Total (n = 132)	Resultado da MAOT		p-value
			Normal (n = 58)	Altered (n = 74)	
**Age**		39.5 ± 12.6	39.0 ± 11.7	40.3 ± 12.8	0.166
**Sex (male)**		85 (64.4%)	38 (70.4%)	47 (60.3%)	0.270
**Survival (years)**		9.456[7.91;10.0]	9.59[7.88;10.0]	9.42[7.84;9.4]	0.342(1)
**MTWA, μV**		76 ± 54.0	32 ± 12.0	110 ± 50.0	**<0.000**
**Primary outcome** **#**		9 (6.8%)	4 (7.0%)	5 (7.0%)	0.824
	SCD	06	02	04	NA
	ICD shock	02	01	01	NA
	SVT	01	01	00	NA
**Echocardiogram, mm**					
	Septum	23.0 [20.0; 27.0]	23.0 [20.0; 27.0]	24.5[20.7; 30.2]	0.532(1)
	Left atrium	42.6 ± 6.99	41.9 ± 7.11	43.2 ± 6.91	0.923
	Left ventricle	4.1 ± 5.39	43.2 ± 4.48	43.1 ± 5.97	0.117
	Ejection fraction, %	72.1 ± 8.07	72.3 ± 7.75	72.1 ± 8.34	0.628
**Obstructive form**		35 (26.5%)	14 (25.9%)	21 (26.9%)	0.531
**SDC risk factors**					
	Syncope	12 (9.1%)	2 (3.7%)	10 (12.8%)	0.073
	NSVT on Holter	37 (28.0%)	9 (16.7%)	28 (35.9%)	**0.016**
	Family history of SCD	27 (20.5%)	7 (13.0%)	20 (25.6%)	0.076
	Septum ≥ 30 mm	27 (20.5%)	3 (5.6%)	24 (30.8%)	**<0.001**
	Fall in SBM during ST	19 (14.4%)	4 (7.4%)	15 (19.2%)	**0.046**
**AHA/ACC 2020** *					
	High risk	70 (53.0%)	22 (28%)	58(72%)	**<0.001**
**Medications**					
	Beta-blockers	95 (72.0%)	44(81.5%)	51 (65.4%)	0.043
	Calcium channel blockers	16 (12.1%)	5 (9.3%)	11 (14.1%)	0.485
	Amiodarone	18 (15.1%)	2 (4.1%)	16 (22.9%)	0.012

(1) Mann-Whitney test, median and interquartile range. NA: Not assessed.

# Primary outcome composed of sudden cardiac death (SCD), resuscitated cardiac arrest, ventricular fibrillation, sustained ventricular tachycardia (SVT), or appropriate implantable cardioverter-defibrillator (ICD) therapy.

* The 2020 AHA/ACC guidelines 2020 defines as high risk those patients with at least one of the following factors: SCD in first-degree relatives aged ≤ 50 years, wall thickness ≥ 30 mm, ≥ 1 episode of unexplained syncope, apical aneurysm, and ejection fraction < 50%. NSVT: non-sustained ventricular tachycardia; SBP: systolic blood pressure; ST: stress test; MTWA: microvolt T-wave alternans.

**Table 2:** Characteristics of patients with primary outcome

Age (years)	Sex	MTWA results	Value (μV)	Risk factors	Indication for ICD#	Survival (years)	Outcome
30	F	Altered	185	0	Class III (B)	1.8	SCD
28	M	Normal	19	0	Class III (B)	3.4	SCD
31	M	Normal	20	Septum≥30 mm	Class IIa (B)	5.3	SCD
24	M	Altered	**56**	TVNS	Class III (B)	6.8	SCD
56	M	Altered	**72**	Septum≥30 mm	Class IIa (B)	6.9	SCD
43	M	Altered	**73**	FH, Septum≥30, NSVT	Class IIa (B)	9.6	ICD shocks
36	F	Normal	27	FH	Class IIa (B)	10.2	SVT
32	F	Altered	**54**	Syncope, HF, NSVT	Class IIa (B)	11.7	SCD
48	M	Normal	14	Syncope	Class IIa (B)	10.2	ICD shocks

# According to 2020 AHA/ACC guidelines; FH: family history of sudden cardiac death; NSVT: non- sustained ventricular tachycardia; SCD: sudden cardiac death; ICD: implantable cardioverter-defibrillator; SVT: sustained ventricular tachycardia; MTWA: microvolt T-wave alternans.

Twelve deaths occurred during the study period, resulting in an overall mortality rate of 1.3% a year; six deaths were from SCD, four from advanced heart failure and two from stroke. Regarding the frequency of primary outcomes, six patients experienced SCD (outside the hospital), two patients had appropriate ICD shocks and one patient had SVT, with a prevalence of 1.0% a year.

Altered MTWA was associated with increased septal thickness, presence of non-sustained ventricular tachycardia (NSVT) by 24-hour Holter monitoring, lower blood pressure levels during exercise and high risk according to the AHA/ACC guidelines.

Different from the expected, MTWA values were lower in the group presenting the primary outcome than the group without events (59 ± 33 μV *versus* 79 ± 42 μV, p=0.352). The vent-free Kaplan-Meier curves were not statistically different between patients with and without altered MTWA (Central Illustration).

MTWA, similar to the AHA/ACC classification, had low accuracy, and was not able to prevent the occurrence of the primary outcome (Table 3).

**Table 3:** Risk, sensitivity, and specificity for the outcome

		Outcome			95%CI					
		Yes	No	OR	Lower	Upper	p	Sensitivity	Specificity	Accuracy
**MTWA**										
	Normal	4	54	0.85	0.21	3.3	0.83	55.6%	43.9%	44.7%
	Altered	5	69							
**2020 AHA guidelines**										
	Low risk	2	60	3.3	0.66	16.8	0.12	77.8%	48.8%	50.8%
	High risk	7	63							

Composite outcome of sudden cardiac death, resuscitated cardiac arrest, ventricular fibrillation or sustained ventricular tachycardia, or appropriate therapy with implantable cardioverter-defibrillator; OR: Odds Ratio MTWA: microvolt T-wave alternans; AHA: American Heart Association guidelines; CI: confidence interval.

## Discussion

The aim of the present study was to assess the prognostic value of MTWA in patients with HCM. Our results showed that patients with HCM had higher MTWA values during the exercise test, with mean of 76±54 μV, as compared with patients with other heart diseases.^20–22^ However, in the present study, altered MTWA test was not associated with the occurrence of potentially fatal ventricular arrythmias in HCM patients.

The increased MTWA values found in our study population may be explained by the presence of an arrhythmogenic substrate typical of HCM. This substrate is composed of myocardial hypertrophy and disarray, associated with formation of a diffuse interstitial fibrosis,^1,23,24^ resulting in repolarization alternans in cardiomyocytes and higher MTWA magnitude.

In addition, the extension of myocardial hypertrophy is associated with an increase in the MTWA magnitude, which was also observed in our population. Altered MTWA was significantly associated with greater septal thickness, corroborating the findings reported by Puntmann et al.^25^

Also, altered MTWA correlates with the reentry and VF.^9–11^ Our results suggest that, in HCM, other arrhythmogenic mechanisms are involved in the cause of ventricular arrhythmias and SCD, since most individuals had high MWTA values, despite a low outcome rate during the follow-up (1.0%/year).

Fuchs et al. ^14^ also reported that MWTA was not useful in predicting SCD. Since MTWA is not a static phenomenon and may change over time, it has been suggested that individuals with normal MWTA values may develop abnormal values over the course of disease.

Today, ICD implantation in HCM is indicated for patients with clinical risk factors and at high risk, following the AHA/ACC guidelines.^3^ In the present study, an altered MWTA was associated with risk factors for SCD and was sensitive in identifying high-risk patients for SCD. However, in our cohort, both MWTA test and AHA/ACC recommendations failed to identify individuals with potentially fatal ventricular arrhythmias or high risk for SCD. Therefore, as also reported by Freitas et al.,^26^ our results demonstrated that the AHA/ACC criteria had low accuracy for detecting individuals who would benefit from ICD. Thus, we believe that further multicentric, nationwide studies are needed, to assess how accurate is the indication for ICD in Brazil, which has been based on results from previous studies conducted in other countries.

### Study limitations

The present study has several limitations. The low event rate during patient follow-up reduces the power of the study, increasing the likelihood of type II error. Single-center studies are subjected to patient selection bias. In addition, some drugs like beta-adrenergic drugs,^17,27,28^ sodium channel blockers^29^ and amiodarone reduce the magnitude of MWTA, and the use of medications was not discontinued before the test in our study. Also, reports on heart rhythm was not available in all cases of SCD, and hence some of these deaths may be attributed to a nonarrhythmic cause.

## Conclusion

The present study showed that altered MTWA was not associated with the occurrence of SCD or malignant ventricular arrhythmias in patients with HCM. The low event rates during patient follow-up corroborate the benign character of this heart disease, with a low mortality rate and a normal life expectancy.
